# Keeping bone resorption markers very low for 1 year after denosumab prevents bone loss at 2 years: the ReoLaus study

**DOI:** 10.1007/s11657-026-01747-0

**Published:** 2026-08-08

**Authors:** Giovanni Liebich, Olivier Lamy, Jérôme Pasquier, Elena Gonzalez-Rodriguez

**Affiliations:** 1 Faculty of Biology and Medicine, University of Lausanne, Lausanne, Switzerland; 2 Interdisciplinary Center for Bone Diseases, Lausanne University Hospital and University of Lausanne, Rue Pierre Decker 4, Lausanne, 1011 Switzerland; 3 Service of Internal Medicine, Lausanne University Hospital and University of Lausanne, Lausanne, Switzerland; 4 Center for Primary Care and Public Health (Unisanté), University of Lausanne, Lausanne, Switzerland; 5 NEXUS Personalized Health Technologies, ETH Zurich, Zurich, Switzerland

**Keywords:** Denosumab discontinuation, BMD evolution, Substitution treatment, Crosslaps threshold, Prediction models

## Abstract

***Summary*:**

BMD loss is common after denosumab discontinuation, and longer treatment is associated with greater BMD loss. Serum crosslaps (sCTX) can predict BMD loss at the lumbar spine after discontinuation. Keeping sCTX below the lower half of the ULN during the first year is needed to avoid BMD loss at 2 years.

**Context:**

After denosumab discontinuation (DD), elevated serum crosslaps (sCTX) is associated with bone mineral density (BMD) loss over 2 years. Bisphosphonates reduce but do not always prevent this loss.

**Objective:**

To define an sCTX threshold below which BMD is maintained after DD.

**Methods:**

Postmenopausal women receiving ≥ 2 denosumab doses and followed ≥ 2 years after DD with regular BMD and sCTX measurements were included retrospectively. Linear regression analysis and ROC curves were performed to define sCTX threshold (%ULN, upper limit of the norm in premenopausal women) preventing 1- and 2-year lumbar spine (LS) BMD loss.

**Results:**

From 161 women who had received 8.0 ± 2.9 injections, 50% lost BMD after 2 years. BMD at DD were similar at all sites in non-losers and losers’ groups but losers had received more injections. Their T-score decreased after 1 and 2 years (*p* < 0.001). BMD loss was highly correlated with increased sCTX during both periods. After DD, first-year sCTX levels predicted 1- and 2-year LS BMD loss. The optimal sCTX thresholds during the first year to avoid LS BMD loss at 1 and 2 years were 69% and 49% ULN; for 100% sensitivity, they were 32% and 23% ULN, respectively. Losers received more often ≥ 3 zoledronate infusions and were less treated with alendronate only.

**Conclusion:**

sCTX level during the first year after DD predict 1- and 2-year LS BMD loss. Keeping sCTX below the lower half of the ULN during the first year is needed to avoid BMD loss. Despite high bisphosphonates doses, it is not always possible to reach this target.

**Supplementary Information:**

The online version contains supplementary material available at 10.1007/s11657-026-01747-0.

## Introduction

Denosumab is a fully human monoclonal antibody with high affinity and specificity for the receptor activator of NF-kB Ligand (RANKL). Mimicking the endogenous soluble receptor osteoprotegerin, it prevents RANKL from binding RANK on osteoclasts, thus inhibiting their differentiation, activation, and function [1]. Long-term denosumab treatment leads to continuous increases in bone mineral density (BMD) and low risk of vertebral and non-vertebral fractures [2–4]. Given its efficacy, safety, and ease of use, denosumab (60 mg subcutaneously every 6 months) has been widely used since 2010 in patients with osteoporosis and for preventing bone loss during long-term glucocorticoid, aromatase inhibitor or androgen inhibitor therapy.

Without bisphosphonate relay, denosumab discontinuation induces a 2-year rebound effect, characterized by increased osteoclast activity [5, 6], elevated bone turnover markers (BTMs), loss of BMD gains [7], and higher risk of multiple spontaneous vertebral fractures (MSVFs) [8–14]. This risk is greater in patients with prior vertebral fractures, those without bisphosphonate (BP) treatment before [12] and especially after denosumab discontinuation [12, 13], and those with longer denosumab treatment [9, 13, 15, 16]. A potent bisphosphonate is thus mandatory after denosumab discontinuation [17–22]. The European Calcified Tissue Society (ECTS) recommends oral bisphosphonate for 1–2 years in low-risk patients treated ≤ 2.5 years and zoledronate for longer treatments or high-risk patients, with BTMs guiding treatment duration [18]. The follow-up of serum crosslaps (sCTX) is recommended in several studies [18, 21, 23]. Sølling et al. propose monitoring of sCTX at 3 and 6 months after the start of replacement treatment with zoledronate/alendronate, with the aim of not exceeding the upper limit of the norm (ULN) before menopause [21]. Other authors propose more aggressive management to maintain sCTX in the lower half of the normal range before menopause [18, 24].

The incidence of new VFs is markedly reduced with BP after denosumab [12], but loss of BMD is almost always present [17, 20, 22, 25, 26]. Targeting low sCTX levels aims not only to prevent fractures but also to preserve BMD gains. Zoledronate, the most studied option, may require more than one infusion to minimize BMD loss [17, 20, 22, 25, 26]. However, no standard consolidation regimen has yet been established to maintain BMD after denosumab.

We present the full 2-year results of the Rebound Effect Observatory study in Lausanne (ReoLaus), a prospective observational study of patients discontinuing denosumab and followed for 2 years.

Preliminary 1-year findings [23] suggested that maintaining sCTX within the lower premenopausal reference range after denosumab may preserve lumbar spine (LS) BMD. We investigated the relationship between sCTX and BMD over the 2-year rebound period, aiming to identify an sCTX cut-off preventing BMD loss. We hypothesized that maintaining sCTX below a specific threshold, through tailored bisphosphonate therapy, would limit BMD decline and fracture incidence.

## Methods

### Setting/study design

ReoLaus is a longitudinal cohort study with reutilization of data collected during routine clinical follow-up at the Interdisciplinary Center of Bone Diseases (CiMO), Lausanne University Hospital [23]. The primary outcomes were as follows: (1) to confirm whether bone turnover markers (BTMs) predict bone mineral density (BMD) loss during the 2-year rebound period and (2) to define a BTMs threshold preventing BMD loss. The secondary outcome was to identify characteristics of patients maintaining BMD (osteoporosis risk factors, treatments, BTMs, and BMD changes) before, during, and after denosumab therapy. For clarity, the end of denosumab therapy/efficacy (defined as 6 months after the last injection) was considered as the reference timepoint corresponding to rebound onset and to the first DXA assessment (T0). Subsequent timepoints were defined relative to T0 (T1 and T2).

### Population

**Fig. 1:**
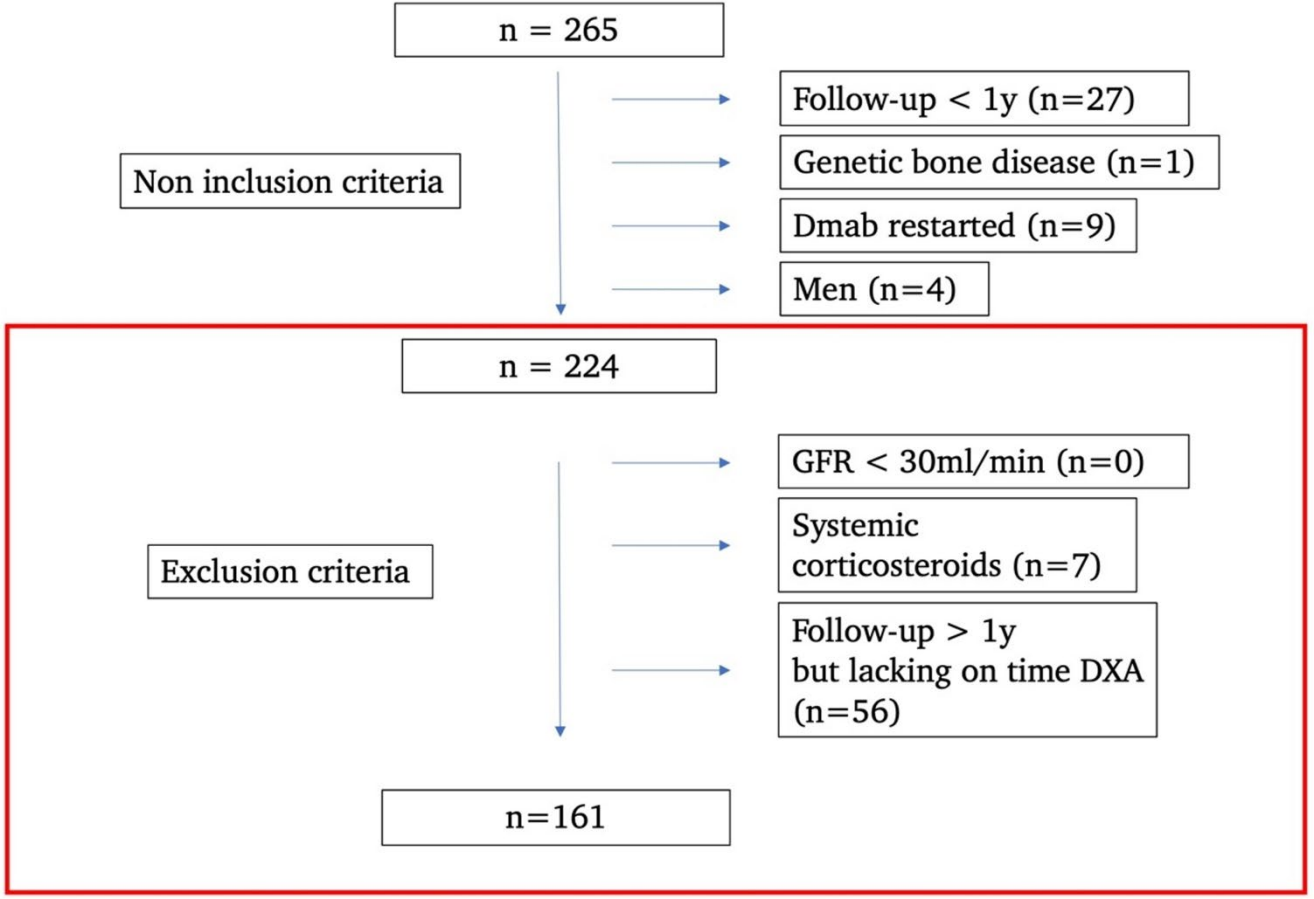
Non-inclusion and exclusion criteria flowchart of the study. Dmab = denosumab; GFR = glomerular filtration rate; systemic corticosteroids (at least 3 months of prednisone equivalent doses of 5 mg or more during or after denosumab); DXA = dual X-ray absorptiometry

Two hundred sixty-five patients treated with denosumab for at least 2 consecutive injections between January 15th, 2011, and June 15th, 2019 (first 15.01.11–15.08.17; last 15.06.14–15.06.19) were reviewed for this study. We included postmenopausal women, who stopped denosumab after receiving at least two injections of 60 mg given regularly every 6 months (± 15 days), followed at our Center for at least 12 months after T0. Treatment could be given for osteoporosis treatment, or for prevention of bone loss under aromatase inhibitors given for breast cancer. Exclusion criteria included glomerular filtration rate < 30 ml/min, systemic corticosteroid therapy during denosumab or its rebound period (prednisone equivalent doses ≥ 5 mg daily for > 3 months), insufficient densitometric data within the established time limits (i.e., patients lacking at least two post-denosumab DXAs (DXA0, DXA1, and DXA2)), or missing clinical information (Fig. 1). One hundred sixty-one postmenopausal women were included.

#### Data collection

Data were collected retrospectively between 1st November 2020 and 31th January 2021 on Excel© sheets (Microsoft) after extraction on the patients records. Data were classified relative to denosumab therapy in three periods: (1) before; (2) during (from initiation to T0, 6 months after the last injection); and (3) after T0 (from 6 months after last injection, to the last DXA measurement for each patient, i.e., T1 or T2).

##### Population characteristics

Age at denosumab initiation, discontinuation, and the menopausal age, as well as anthropometric characteristics (height, weight, and BMI at denosumab initiation), were collected. Major osteoporotic fractures (vertebral, femoral, pelvis, and proximal humerus), other osteoporotic fractures (wrist, ribs, and upper tibia), and atraumatic non-typical osteoporotic fractures (tibial, peroneal, stress fractures, and others) before, during, and after denosumab treatment were analyzed. For vertebral fractures during denosumab, sporadic fractures were differentiated from rebound fractures, which occurred after an accidental delay of > 6 months between two injections. Fractures count ended 24 months after T0 (30 months after last injection). We classified osteoporosis risk factors in two groups: aromatase inhibitors (≥ 3 months therapy) and other risk factors. Due to their low prevalence, the other risk factors were grouped together and included long-course systemic corticosteroid therapy (≥ 3 months of prednisone equivalent doses of ≥ 5 mg before denosumab), malabsorption syndrome (celiac disease, gastric bypass, and bowel inflammatory diseases), primary hyperparathyroidism, rheumatoid arthritis, multiple sclerosis, monoclonal gammopathy, and systemic mastocytosis.

##### Treatment

Pre-denosumab therapies included bisphosphonates, teriparatide, strontium ranelate, and previous denosumab. Duration and interval between treatments were recorded. Post-denosumab therapy was at physicians’ discretion and mainly consisted of zoledronate infusions (5 mg) or oral weekly alendronate (70 mg). Other treatments (teriparatide or ibandronate) were chosen for selected patients. Type and time of each substitution therapy were analyzed up to the last DXA examination.

##### Bone turnover markers

Fasting serum C-terminal telopeptide (sCTX) and procollagen type 1 N-terminal propeptide (P1NP) were measured: (1) before (in the 12 months before the first denosumab injection); (2) during (one in the 12 months after the first denosumab injection, one at the time of the last denosumab injection and 3 months later); and (3) as frequently as possible after T0 (since 6 months after the last denosumab injection). sCTX values were measured in early morning fasting blood samples at the central clinical routine laboratory of the Lausanne University Hospital in more than 90% of cases (sCTX premenopausal women normal ranges: 25–573 ng/l). As reference values may differ from laboratory to laboratory, we expressed the results in percent of the premenopausal ULN (mostly 573 ng/L for sCTX and 58.6 μg/L for P1NP). BTMs were collected in monthly intervals (± 15 days), with a variable number of measurements per patient during the rebound period (approximately 4 measurements per patient-year), depending on patient availability (i.e., the number of follow-up visits).

##### Bone mineral density

BMD was measured with dual-energy X-ray absorptiometry (DXA) on different machines. Most of the examinations were performed at the Lausanne University Hospital on two different machines (GE Lunar or Hologic). For each patient, specific parameters were maintained during the follow-up: (1) femoral neck (FN) and total hip (TH) were measured on the same side; (2) the same vertebrae were chosen for the LS analysis, after exclusion of fractured vertebrae; (3) only TBS values measured always on the same machine were considered, due to the fact that TBS values differ between the DXA machines. To standardize measurements, BMD results were expressed in T-scores, as a number of patients had to switch DXA machine during follow-up.

DXA-1 (first) was taken before denosumab (12 months before to 3.5 months after the first injection). DXA0 (baseline) corresponds to the end of denosumab efficacy (T0, 12 months before to 3.5 months after). DXA1 was taken 12 months (8.5 to 15.5), and DXA2 24 months (20.5 to 36), respectively, after T0. Follow-up ended at each patient’s last DXA.

Means and SDs of *T*-scores and TBS were calculated at each time point. Mean differences in *T*-scores and their SDs were computed for the intervals: (DXA0)–(DXA-1), (DXA1)–(DXA0), (DXA2)–(DXA1), and (DXA2)–(DXA0), and annualized changes were standardized by time intervals.

#### Statistical analysis

Baseline characteristics were summarized using means and standard deviations (SDs) for continuous variables, while frequencies and proportions for categorical variables. Statistical comparisons were made between included and excluded participants, and between groups. Welch’s t-tests were used to compare continuous variables, and Fisher’s exact tests were used for categorical variables.

##### Group definition

A least significant change (LSC) of ± 0.2 SD was set for LS *T*-score, of ± 0.3 SD for FN and TH *T*-scores, and of ± 3.8% for TBS, between two DXA time-points. LSC thresholds were based on precision error established at our center and used to define a significant difference between two measurements at each site. Patients were classified as “losers” or “non-losers” based on an annualized reduction in LS BMD *T*-scores (≥ 0.2 SD loss) between T0 (6 months after the last injection) and T2 (2.5 years after the last injection). To further explore the definition of “losers”, subgroup analyses were conducted.

##### Association between sCTX AUC and BMD T-score changes

sCTX values at T0, T1, and T2 were estimated using the following rules: (1) if measurements existed at T0, T1, or T2, the corresponding CTX values were used; (2) if no exact measurement was available, values were interpolated using linear interpolation when surrounding data points were available, defined as the presence of observations both before and after the target timepoint; (3) in cases where measurements were sparse or outside the interpolation range (i.e., when observations were available only on one side of the target timepoint or a single measurement was available), values were set to NA (non-assessed). No extrapolation beyond the observed data was performed.

sCTX AUC values were calculated for the intervals T0-T1, T1-T2, and T0-T2, standardized as follows: (1) AUC01/(T1-T0); (2) AUC12/(T2-T1); and (3) AUC02/(T2-T0). Linear regression models were used to assess the association between sCTX AUC and BMD *T*-score changes.

##### Prediction models

ROC analyses evaluated the predictive value of sCTX AUC for identifying “losers.” The predictor was sCTX AUC, and “loser” status was defined by BMD reductions at LS, FN, TH, or TBS. Thresholds for sCTX AUC were determined using the Youden index to maximize sensitivity and specificity. Figures illustrate the linear regression and threshold analyses, highlighting key findings in terms of sensitivity, specificity, positive predictive value (PPV), and negative predictive value (NPV).

#### Informed consent

Informed consent was obtained from all participants. The study was approved by the institutional ethics committee (CER-VD 2017–02135) and extended in July 2019 (Amendment 1) for the second study year.

## Results

### Population characteristics

The characteristics of the 161 included and the 63 excluded postmenopausal women were very similar (Table 1) except for the denosumab treatment duration and the percentage of women who received a bisphosphonate after denosumab, both higher in included patients. The included women discontinued denosumab after a mean of 8.0 ± 2.9 injections at a mean age of 65.8 ± 9.5. Seventy-seven patients (48%) had fragility fractures before the beginning of denosumab, of whom 49 (30%) vertebral fractures. Thirty participants (19%) were treated with aromatase inhibitors. Sixty patients (37%) were treated with bisphosphonate before denosumab, while 152 (94%) were treated with bisphosphonate 2.0 ± 3.2 months after T0. DXA1 and DXA2 were made respectively at 1.0 ± 0.2 and 2.1 ± 0.2 years after T0. Supplementary Table 1 shows average BMD evolution at each site. At all sites, mean BMD increased during denosumab. During the first year after discontinuation, mean BMD decreased at all sites. During the second year, mean BMD remained stable at all sites.

sCTX levels were elevated before denosumab, decreased during denosumab, and increased at 1, 2, 7, 12, and 24 months after T0 (Table 1). At these different time points, the results were available for 27 to 81 women.

**Table 1:** Characteristics of the included and excluded patients

	Included patients (*n* = 161)	Excluded patients (*n* = 63)	*p*-value
Age at dmab initiation, years	65.8 ± 9.5	64.8 ± 11.0	0.52
Age at menopause, years	49.5 ± 4.4	48.4 ± 4.7	0.16
BMI, kg/m^2^	23.3 ± 3.5	23.9 ± 5.3	0.47
Fractured patients, *n* (%)			
Before dmab	77 (48)	28 (44)	0.65
Vertebral	49 (30)	14 (22)	0.22
During dmab	11 (7)	9 (14)	0.08
Vertebral	5 (3)	2 (3)	0.98
After dmab	10 (6)	5 (8)	0.64
Vertebral	9 (6)	4 (6)	0.83
Aromatase inhibitors, *n* (%)	30 (19)	18 (29)	0.10
Other risk factors, *n* (%)	23 (14)	15 (24)	0.09
Patients under bone treatment before dmab, *n* (%)	60 (37)	31 (50)	0.14
Number of dmab injections	8.0 ± 2.9	6.8 ± 3.4	0.01
Patients under BP after dmab, *n* (%)	152 (94)	53 (84)	0.01
Delay 6 months after last dmab, months	2.0 ± 3.2	3.8 ± 5.0	0.16
Duration BP treatment until DXA1, months	10.9 ± 2.3	11.1 ± 3.5	0.84
Duration BP treatment until DXA2, months	23.9 ± 4.2	24.5 ± 5.8	0.74
T-score LS, SD (n)			
DXA-1, before dmab	−2.6 ± 1.1 (117)	−2.5 ± 1.1 (49)	0.46
DXA0	−1.7 ± 1.1 (142)	−1.8 ± 1.1 (50)	0.55
DXA1	−2.0 ± 1.3 (132)	−2.0 ± 1.2 (46)	0.95
DXA2	−2.0 ± 1.2 (128)	−2.2 ± 1.2 (32)	0.57
*T*-score FN, SD (*n*)			
DXA-1, before dmab	−1.9 ± 0.7 (113)	−1.8 ± 0.8 (49)	0.36
DXA0	−1.6 ± 0.8 (135)	−1.8 ± 0.9 (49)	0.36
DXA1	−1.8 ± 0.8 (130)	−2.0 ± 0.8. (42)	0.57
DXA2	−1.7 ± 0.7 (127)	−2.0 ± 0.6 (31)	0.19
*T*-score TH, SD (*n*)			
DXA-1, before dmab	−1.6 ± 0.7 (116)	−1.4 ± 1.2 (51)	0.25
DXA0	−1.4 ± 0.8 (142)	−1.6 ± 0.9 (51)	0.18
DXA1	−1.6 ± 0.8 (131)	−1.8 ± 0.9 (44)	0.49
DXA2	−1.5 ± 0.8 (128)	−1.6 ± 1.0 (31)	0.82
TBS			
DXA-1, before dmab	1.222 ± 0.104 (80)	1.242 ± 0.109 (28)	0.42
DXA0	1.294 ± 0.092 (127)	1.325 ± 0.071 (44)	0.07
DXA1	1.301 ± 0.084 (126)	1.252 ± 0.121 (43)	0.22
DXA2	1.300 ± 0.080 (125)	1.312 ± 0.054 (32)	0.47
sCTX (% ULN)			
Before dmab	92.2 ± 37.4 (81)	78.3 ± 35.9 (27)	0.09
During dmab	9.6 ± 9.0 (81)	8.8 ± 5.0 (23)	0.57
1 m after T0	50.3 ± 46.9 (81)	47.2 ± 34.4 (27)	0.72
2 m after T0	78.1 ± 56.6 (58)	46.5 ± 30.3 (20)	0.003
7 m after T0	81.0 ± 51.5 (52)	72.8 ± 42.9 (17)	0.52
12 m after T0	69.8 ± 41.4 (42)	46.2 ± 29.0 (16)	0.02
24 m after T0	58.9 ± 23.3 (27)	44.9 ± 23.6. (8)	0.17

DXA-1 was taken before the denosumab initiation (between 12 months before and 3.5 months after the first denosumab); DXA0 was taken at T0 (between 12 months before and 3.5 months after the end of denosumab efficacy); DXA1 12 months after T0 (8.5 to 15.5), and DXA2 24 months after T0 (20.5 to 36); other risk factors include long course systemic corticosteroid therapy (at least 3 months of prednisone equivalent doses of 5 mg or more before denosumab), malabsorption syndrome (celiac disease, gastric bypass, and bowel inflammatory diseases), primary hyperparathyroidism, rheumatoid arthritis, multiple sclerosis, monoclonal gammopathy, and systemic mastocytosis. Values are expressed in number (%) or mean ± standard deviation (minimum, maximum)

*Dmab* denosumab; *BMI* body mass index; *BP* bisphosphonate; *LS* lumbar spine; *FN* femoral neck; *TH* total hip; *SD* standard deviation; *m* month(s); *T0* time of rebound effect onset (6 months after last dmab injection); *DXA* Dual X-ray absorptiometry; *TBS* trabecular bone score; *sCTX* serum crosslaps; *%ULN* percentage of the upper limit of the norm for the pre-menopausal women

### Characteristics of losers and non-losers

Table 2 shows the characteristics of the included patients. Half were losers (ΔLS T-score ≤ −0.2 SD between DXA2 and DXA0) and half non-losers. Risk factors, number of fractured patients at all time points, and previous treatments were similar between groups. Patients in the losers’ group received significantly more denosumab injections. Two years after T0, losers have a worse LS *T*-score; *T*-score at other sites were not significatively different. Considering the entire two-year period there was a change on LS values of −0.5 ± 0.3 SD in the losers’ group vs. + 0.1 ± 0.3 SD in the non-losers’ group (difference 0.6 ± 0.4 SD; *p* < 0.001). BMD change patterns differed: losers’ group lost BMD in both years, while the non-losers’ group lost in the first year and gained in the second. sCTX AUC was significantly higher in the losers each year and during the whole period (Table 2), while P1NP AUC did not differ (results not shown).

**Table 2:** Characteristics of losers and non-losers at lumbar spine 2 years after the end of denosumab efficacity (T0)

LS 02, *n* = 114	Losers, *n* = 57	Non-losers, *n* = 57	*t*-test
	Mean ± SD	Mean ± SD	***p***-value
Age			
dmab initiation, years	63.7 ± 7.9	66.5 ± 10.7	0.11
dmab end, years	68.4 ± 8.2	70.3 ± 10.4	0.26
Time from menopause to dmab, years	13.1 ± 7.7	15.3 ± 9.2	0.23
BMI, kg/m^2^	23.0 ± 3.5	23.7 ± 3.2	0.26
Dmab			
Injections, number	9.0 ± 2.8	7.3 ± 2.9	0.002
Duration, months	57.1 ± 18.6	46.3 ± 18.4	0.002
DXA0			
TS LS, SD	−1.7 ± 1.3	−1.8 ± 1.0	0.50
TS FN, SD	−1.7 ± 0.7	−1.6 ± 0.8	0.32
TS TH, SD	−1.4 ± 0.7	−1.3 ± 0.8	0.41
DXA1			
TS LS, SD	−2.2 ± 1.4	−2.0 ± 1.1	0.55
TS FN, SD	−1.9 ± 0.7	−1.7 ± 0.7	0.10
TS TH, SD	−1.7 ± 0.7	−1.4 ± 0.8	0.07
DXA2			
TS LS, SD	−2.3 ± 1.3	−1.8 ± 1.1	0.03
TS FN, SD	−1.9 ± 0.8	−1.6 ± 0.7	0.05
TS TH, SD	−1.6 ± 0.8	−1.4 ± 0.8	0.12
DXA changes 01 (first year)			
ΔSD LS	−0.4 ± 0.4	−0.1 ± 0.4	< 0.001
ΔSD FN	−0.2 ± 0.2	−0.1 ± 0.2	0.12
ΔSD TH	−0.2 ± 0.2	−0.1 ± 0.3	0.12
DXA changes 12 (second year)			
ΔSD LS	−0.1 ± 0.4	0.2 ± 0.3	< 0.001
ΔSD FN	−0.0 ± 0.2	0.1 ± 0.2	0.04
ΔSD TH	0.0 ± 0.3	0.1 ± 0.4	0.39
DXA changes 02 (2 years)			
ΔSD LS	−0.5 ± 0.3	0.1 ± 0.3	< 0.001
ΔSD FN	−0.1 ± 0.3	−0.0 ± 0.3	0.07
ΔSD TH	−0.2 ± 0.3	−0.1 ± 0.3	0.06
sCTX 01 (first year)			
AUC (%ULN)	79.1 ± 35.2	63.6 ± 41.4	0.04
sCTX 12 (second year)			
AUC (%ULN)	66.7 ± 20.4	56.1 ± 28.3	0.03
sCTX 02 (2 years)			
AUC (%ULN)	73.2 ± 24.2	60.2 ± 32.0	0.03
	Losers	Non-losers	Fischer
	*n *(%)	***n ***(%)	***p***-value
Risk factor			
Aromatase inhibitors, *n* (%)	8 (14)	14 (25)	0.24
Other risk factors, *n* (%)	6 (11)	13 (23)	0.13
Fractured patients			
Before dmab, *n*	28 (49)	25 (44)	0.71
Vertebral fractures, n	17 (30)	16 (28)	1.00
During dmab, *n*	4 (7)	3 (5)	1.00
Vertebral fractures, *n*	2 (4)	2 (4)	1.00
After dmab, *n*	3 (5)	3 (5)	1.00
Vertebral fractures, *n*	3 (5)	2 (4)	1.00
Osteoporosis treatment after dmab			
BPs, *n*	56 (98)	54 (95)	0.72
Alendronate (ALN) only, *n*	1 (2)	8 (14)	0.03
Zoledronate (ZOL) only, *n*	36 (63)	32 (56)	0.57
ALN & ZOL mixed, *n*	19 (33)	11 (19)	0.14
1*ZOL, *n*	20 (35)	17 (30)	0.69
2*ZOL, *n*	19 (33)	24 (42)	0.44
≥ 3*ZOL, *n*	16 (28)	4 (7)	0.006
Time from end dmab to BP, months	2.6 ± 4.1	1.7 ± 2.1	0.13 (*t*-test)
Osteoporosis treatment before dmab			
BPs, *n*	16 (28)	27 (47)	0.05
ALN, *n*	7 (12)	15 (26)	0.10
ZOL, *n*	2 (4)	5 (9)	0.44
Two or more different BPs, *n*	1 (2)	4 (7)	0.36
BPs duration, months	55.4 ± 52.2	65.5 ± 45.8	0.52 (*t*-test)
BP before & after	15 (26)	25 (44)	0.08
Complementary treatment during dmab	5 (9)	6 (11)	1.00

DXA0 was taken at T0 (between 12 months before and 3.5 months after the end of denosumab efficacy); DXA1 12 months after T0 (8.5 to 15.5), and DXA2 24 months after T0 (20.5 to 36); 365-days standardized DXA changes expressed in absolute t-score SD differences (ΔSD) for each site; other risk factors include: long course systemic corticosteroid therapy (at least 3 months of prednisone equivalent doses of 5 mg or more before denosumab), malabsorption syndrome (celiac disease, gastric bypass, bowel inflammatory diseases), primary hyperparathyroidism, rheumatoid arthritis, multiple sclerosis, monoclonal gammopathy and systemic mastocytosis. Values are expressed in number (%) or mean ± standard deviation

*Dmab* denosumab; *BMI* body mass index; *BPs* bisphosphonates; *ALN* alendronate; *ZOL* zoledronate; *TS* t-score; *LS* lumbar spine; *FN* femoral neck; *TH* total hip; *SD* standard deviation; m month(s); *T0* time of rebound effect onset (6 months after last dmab injection); *DXA* Dual X-ray Absorptiometry; *01* first year; *12* second year; *02* 2 years; *TBS* Trabecular bone score; *sCTX* serum crosslaps; *AUC* area under the curve; *%ULN* percentage of the upper limit of thenorm for the pre-menopausal women

After denosumab, nearly all patients received bisphosphonates (98% of losers vs. 95% of non-losers; *p* = 0.72). The time from the end of denosumab treatment (T0) to bisphosphonate initiation was similar (2.6 ± 4.1 months in losers and 1.7 ± 2.1 months in non-losers (*p* = 0.13)). Bisphosphonates regimen was not standardized, as it was guided by bone turnover markers and clinical judgment. Alendronate only was more frequent in non-losers (14% vs. 2%; *p* = 0.03), while zoledronate only and sequential therapy of alendronate and zoledronate were similar between groups. The number of zoledronate infusions was generally comparable, except that three or more zoledronate infusions were more common in the loser group (28% vs. 7%; *p* = 0.006). A non-significant trend shows a lower percentage of losers’ group patients treated with bisphosphonates prior to denosumab (28% vs. 47%, *p* = 0.05).

### Bone turnover markers and bone mineral density association

**Fig. 2:**
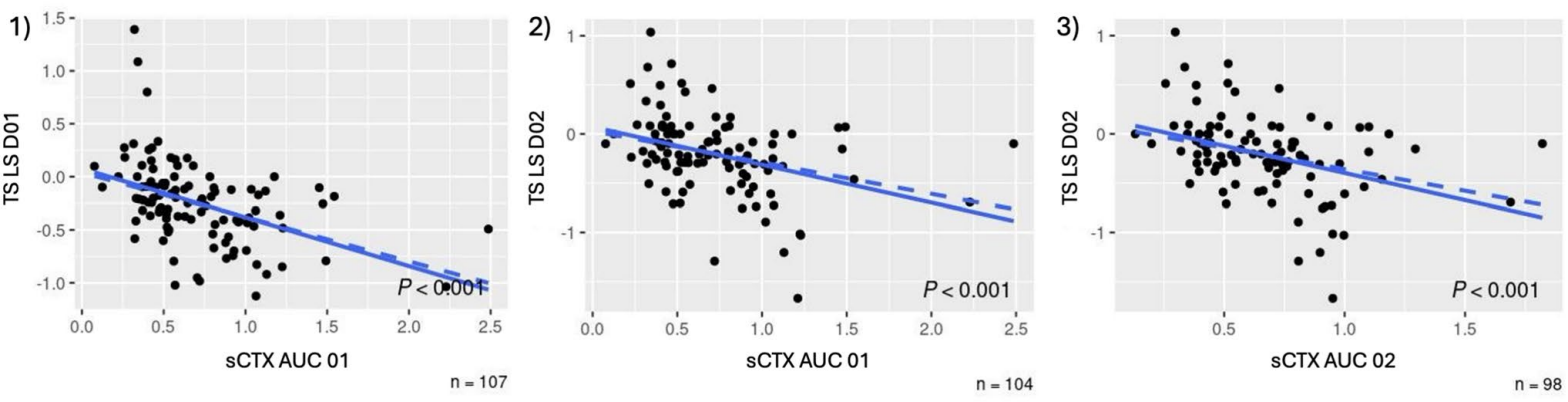
Linear regression (LR) graphs: lumbar spine BMD changes and serum crosslaps AUC. (**1**) LR between 365-day standardized *T*-score change on lumbar spine during the first year after T0 (*Y*-axis) and 365-day standardized sCTX AUC during the first year after T0 (6 months after last denosumab injection) (*X*-axis). (**2**) LR between 730-day standardized *T*-score change on lumbar spine during the 2 years after T0 (*Y*-axis) and 365-day standardized sCTX AUC during the first year after T0 (*X*-axis). (**3**) LR between 730-day standardized *T*-score change on lumbar spine during the 2 years after T0 (*Y*-axis) and 730-day standardized Cross Laps AUC during the 2 years after T0 (*X*-axis)

Linear regression showed a strong association between LS T-score loss in the first year and higher sCTX AUC at any analyzed period (Fig. 2). Instead, the LS T-score during the second year is relatively stable and not influenced by sCTX during the same period, indicating that most BMD loss occurred in the first rebound year and was driven by increased bone turnover.

**Fig. 3:**
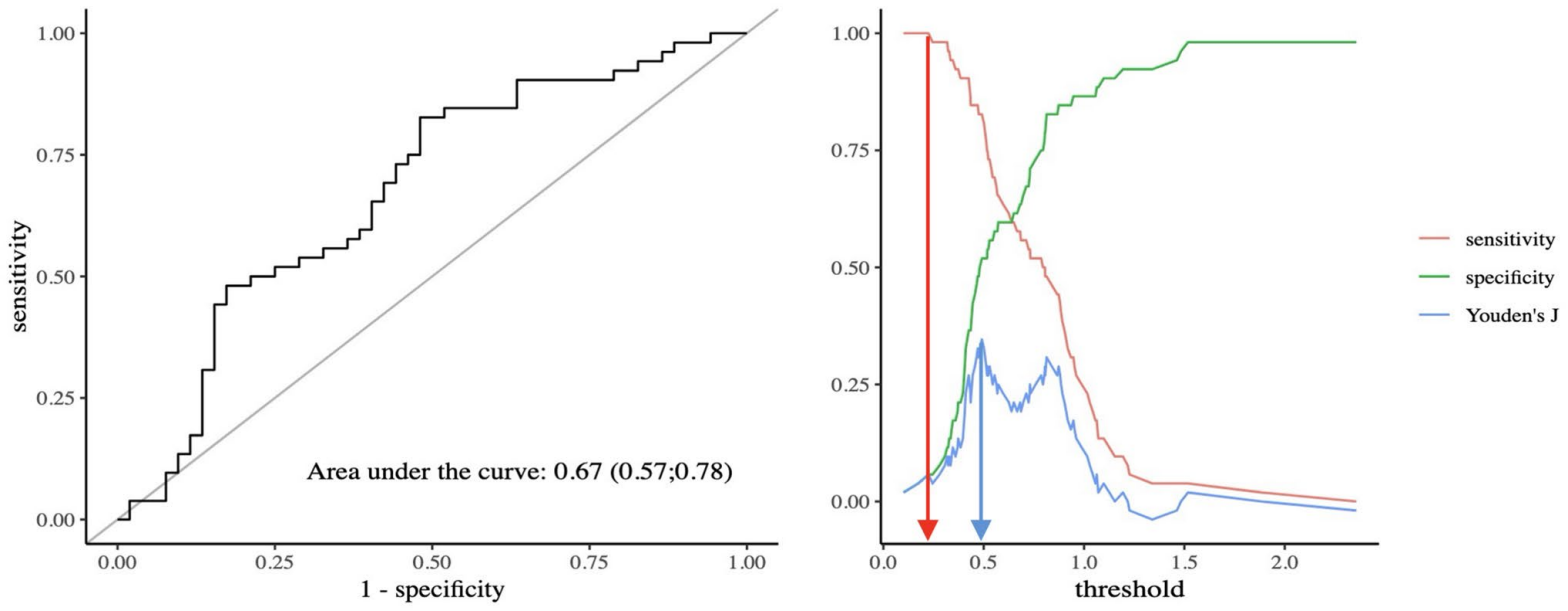
ROC curve of sCTX AUC during the first year predicting lumbar spine loss at 2 years and threshold definition. Relationship between lumbar spine loss at 2 years after T0 and sCTX AUC during the first year after T0 (ROC curve (left), sensitivity-specificity-Youden threshold graph (right). Threshold values expressed in proportion of the ULN for the pre-menopausal women. The red arrow shows the 100% sensitivity threshold (0.23*ULN—129 ng/L); the blue arrow shows the optimal sensitivity-specificity-Youden threshold (0.49*ULN—281 ng/L)

Table 3 and Fig. 3 present Youden-index-derived cut-offs defining sCTX thresholds maximizing the sensitivity and specificity in some selected ROC models to avoid bone loss (n positive > 10 to reduce the overfitting risk, ROC AUC > 0.65). To prevent the LS BMD loss after one year, we must maintain sCTX < 0.69 the ULN, which corresponds to a cut-off of 397 ng/L considering an ULN of 573 ng/L. For a 100% sensitivity (no patient loses BMD), the cut-off is 185 ng/L. Considering the sCTX AUC during the first year, a cut-off < 0.49 the ULN (281 ng/L) avoids LS BMD loss at 2 years; for a 100% sensitivity, the cut-off is 129 ng/L (Fig. 3). The results are similar for the sCTX AUC during the 2 years to avoid LS BMD loss at 2 years. Table 3 lists corresponding thresholds for TH, FN or a combination of LS and TH.

**Table 3:** Prediction models: sCTX AUC predictor for BMD loss response

Response	sCTX AUC predictor	nobs	npos	ROC AUC (CI)	Youden’s index	sCTX cut-off maximizing Youden’s index		sCTX cut-off at 100% sensitivity	
						%ULN	Value (ng/L)	%ULN	Value (ng/L)
LS Loss 01	01	107	59	0.686 (0.584–0.789)	0.330	69.3	397.1	32.2	184.5
TH Loss 01	01	106	25	0.676 (0.568–0.783)	0.367	48.0	275.0	34.0	194.8
FN Loss 01	01	102	15	0.789 (0.657–0.922)	0.577	66.7	382.2	24.1	138.1
LS Loss 02	**01**	**104**	**52**	**0.674 (0.569–0.779)**	**0.346**	**49.1**	**281.3**	**22.5**	**128.9**
TH Loss 02	01	102	21	0.746 (0.630–0.862)	0.490	80.2	459.5	40.0	229.2
FN Loss 02	01	101	22	0.739 (0.629–0.850)	0.391	80.2	459.5	40.0	229.2
LS & TH Loss 01	01	104	18	0.718 (0.606–0.829)	0.395	48.0	275.0	48.0	275.0
LS or TH Loss 01	01	109	66	0.689 (0.583–0.795)	0.357	58.6	335.8	32.2	184.5
LS Loss 02	02	98	50	0.678 (0.569–0.788)	0.347	63.8	365.6	35.5	203.4
TH Loss 02	02	96	19	0.779 (0.664–0.894)	0.515	86.0	492.8	39.3	225.2
FN Loss 02	02	95	20	0.739 (0.632–0.847)	0.427	69.3	397.1	48.2	276.2
LS & TH Loss 02	02	98	13	0.755 (0.621–0.888)	0.500	65.8	377.0	39.3	225.2
LS or TH Loss 02	02	96	56	0.734 (0.628–0.841)	0.421	63.8	365.6	35.5	203.4

Prediction models are ranked for sites and time of the response (LS, FN, TH, LS and TH, LS, or TH; first and second years) and for the time of the predictor sCTX AUC (first and second years). sCTX cut-off are expressed in % of the upper limit of the norm (ULN) and in ng/L considering a ULN of 573 ng/L at the maximum of Youden’s index and at 100% sensitivity

*nobs* number observation; *npos* number positive (losers); *ROC AUC* area under the curve for the ROC curve (confidence interval); *FN* femoral neck; *LS* lumbar spine; *TH* total hip; *01* first year; *12* s year; *02* 2 years

## Discussion

These extended results of the ReoLaus cohort show that 50% of the women retained the BMD gained under denosumab 2 years after stopping it. In those who lost BMD, most of the loss took place during the first year. Maintenance of BMD at LS was associated to a shorter denosumab treatment and favored by keeping sCTX values in the lower half of the premenopausal range, independently of the intensity of the bisphosphonates treatment given after denosumab.

In our study, 50% of patients maintained LS BMD 2 years after denosumab discontinuation. This is similar to both the observational study from Grassi et al. with 61% patients losing BMD over the LSC at the LS during the 17 months follow-up after denosumab discontinuation [24], and the randomized controlled trial from Sølling et al. [27] in which 36% (21/58) lost LS BMD at 2 years. However, comparison of different studies is hampered by the use of different criteria to define BMD loss, and different bisphosphonates replacement protocols used.

Consistent with a systematic review by Tsourdi et al. [18], longer denosumab treatment leads to greater post-discontinuation loss both in patients without and with antiresorptive drugs replacement (mostly bisphosphonates). Loss of the BMD gained despite bisphosphonates therapy is mostly observed in patients who have received more than 3 years of denosumab as compared to shorter treatments [20, 24, 28, 29]. Two observational studies showed that patients receiving ≤ 6 denosumab injections and being switched to zoledronate maintained LS BMD, while those with longer treatment lost part of the BMD gained under denosumab [28, 29]. Consistently, in our study patients losing BMD at LS had been treated for a longer period with denosumab, with mean duration in non-losers’ group around 3.5 years (7.3 injections, vs. 9.0 in losers’ group, *p* = 0.002).

Multiple studies have explored other risk factors linked to stronger BMD loss: younger age [20, 29, 30], greater BMD gain during denosumab treatment [24, 29], absence of prior BPs [29], or higher sCTX before denosumab treatment [24]. However, there is strong inconsistency in the significance of these factors between studies. In our study, there were no significant differences between groups, although a non-significant trend was observed for prior bisphosphonate use.

Higher BMD loss after longer denosumab treatment seems to be due to a stronger bone remodeling activity, as estimated by the bone remodeling markers. Indeed, although bone markers are kept low on long-term denosumab, Eastell et al. shows that the longer the time since the initial dose, the higher the level of sCTX at 6 ± 1 months (treatment by time since injection interaction, *p* < 0.0001) [31]. We calculated the AUC values of sCTX during the first, the second, and both years after denosumab discontinuation. All were in the premenopausal range, but they were significatively higher at each period in losers’ group than in non-losers, consistent with a stronger rebound in patients losing BMD. This is similar to the study by Grassi et al. [24], who compared sCTX at denosumab discontinuation and after 6 months in patients losing or maintaining BMD.

The stronger rebound after longer denosumab treatment manifests also, in the absence of subsequent treatment, with more patients presenting with multiple vertebral fractures [13], a higher number of fractures per patient [9], and earlier occurrence after denosumab interruption [15]. Multiple studies showed that bisphosphonates after denosumab discontinuation strongly decrease the risk of rebound vertebral fractures [12, 18, 19]. To our knowledge, no study analyzed whether BMD loss despite bisphosphonate treatment is associated to vertebral fractures. Despite 94% of the patients benefiting of bisphosphonate replacement, 6% of patients experienced fractures during the rebound period, with 9 out of 10 being vertebral fractures, without significant differences between the losers’ and the non-losers’ groups, but numbers are low. Nine of the 10 patients who experienced fractures were treated with bisphosphonates before the occurrence of fracture. No MSVFs were observed under substitutive treatment suggesting a positive effect of BPs substitution on preventing MSVFs.

In our study, BMD loss in losers’ group occurred in both years, but mainly in the first (−0.4 ± 0.4 SD, vs. −0.1 ± 0.4 SD in the second). In the non-losers, the slight first-year loss was compensated by a second-year gain (−0.1 ± 0.4 SD and + 0.2 ± 0.3 SD, respectively). Group differences remained significant regardless of period. Although in studies analyzing different periods after denosumab discontinuation main loss occurs during the first year, kinetics seem to be slightly different. In a subgroup analysis from the Everts-Graber study, there were no significant BMD changes between 12 and 42 months of follow-up, indicating that the majority of bone loss occurred in the first months after denosumab discontinuation [25]. Similarly, Sølling et al. showed that in a group of patients followed for 2 years, BMD was stable in the second year after the zoledronate infusion [20]. Another study showed continued loss in the first year and thereafter [30]. However, in these studies there was no separate analysis of patients maintaining or loosing BMD. In our whole population, without subgroup analysis, the pattern was consistent with Everts-Graber’s findings [25] with LS BMD loss limited to the first year (−0.3 ± 0.4 SD) and stability in the second (+ 0.0 ± 0.4 SD, Supplementary Table 1).

As in Grassi et al. [24], BMD evolution after denosumab was directly associated with sCTX levels. During the first year, the sCTX cut-offs linked to BMD maintenance were similar to those in Grassi et al. with an optimal threshold to avoid LS BMD loss after 17 months of 453 ng/L (sensitivity 78%, specificity 67%), similar to our cut-off of 397 ng/L over 2 years. For a 100% sensitivity, they found a value of 212 ng/L as compared to 185 ng/L in our study. Importantly, results are similar despite the significant differences between the studies. First, patients have been managed very differently. Grassi et al. followed a standardized protocol with a first zoledronate perfusion 6 months after last denosumab injection, that was repeated after 6 months if sCTX > 280 ng/L (threshold proposed in the ECTS position statement on the management of denosumab discontinuation [18]) based on mean sCTX values in healthy premenopausal women in the US [32]. Our patients were mostly treated following our first recommendations on denosumab discontinuation published in 2019 [33] in which alendronate is an alternative and multiple zoledronate perfusions are possible. Second, while their sCTX threshold is based on a single sCTX collection at 6 months [24], we calculated a sCTX AUC based on multiple dosing during each year.

Indeed, the optimal treatment regimen to avoid BMD loss after discontinuation has not yet been established. The ECTS proposed in 2020 different approaches: zoledronate infusion after long-term (> 2.5 years) and weekly oral alendronate after short-term (≤ 2.5 years) denosumab treatment [18]; repeated zoledronate perfusions are proposed in case of uncontrolled sCTX values or BMD loss during follow-up, if measured. Notably, patients loose BMD despite very different intensity of bisphosphonate replacement in different studies: while Grassi et al. gave a maximum of 2 zoledronate perfusions 6 months apart considering a low sCTX threshold [24], in our study 20 patients benefited of 3 or more perfusions as they had repeatedly higher sCTX values during follow-up. Moreover, losers more frequently required multiple zoledronate infusions (28% vs. 7% of non-losers; *p* = 0.006), and more patients in the non-losers’ group were managed with alendronate alone (14% vs. 2%, *p* = 0.03), suggesting some patients experience a stronger rebound that current protocols cannot fully control to avoid BMD loss.

An additional noteworthy finding is the absence of a significant association between sCTX levels and BMD changes during the second year after denosumab discontinuation at the lumbar spine, in contrast to the significant associations observed between first-year sCTX AUC and first-year BMD loss, first-year sCTX AUC and 2-year BMD loss, as well as two-year sCTX AUC and overall 2-year BMD loss. However, group-based analyses showed that patients classified as BMD losers consistently exhibited higher sCTX AUC values across all timepoints, including the second year. This suggests a persistently higher bone turnover profile in these patients, potentially reflecting an intrinsic predisposition beyond the initial rebound phase. Nevertheless, BMD loss during the second year remained limited, even among losers, likely reflecting the effect of antiresorptive treatment initiated after denosumab discontinuation, and indicating that mechanisms other than active bone resorption may contribute to residual changes in bone density.

### Strengths and limitations

Our study’s strength lies in the cohort size with multiple sCTX measurements over 2 years of follow-up after denosumab discontinuation, making it the most comprehensive study to date of the association between sCTX levels and BMD changes throughout the rebound effect. Frequent sCTX monitoring, combined with the use of the annualized AUC curve, provides a precise assessment of marker trends, significantly strengthening predictive models.

Limitations include the retrospective observational design, which may introduce selection bias and limit causal inference. As this is a homogeneous cohort (Swiss, female) from a single center, its external validity is limited. Moreover, the timing and frequency of sCTX measurements were not standardized, potentially affecting consistency and reliability; we sought to mitigate this by using an ULN-normalized, annualized AUC. The long study period, during which changes in treatment protocols and variability in patient management due to the involvement of multiple healthcare providers may have influenced the outcomes, potentially confounds the findings.

An additional limitation relates to the use of *T*-scores rather than absolute BMD values to estimate longitudinal changes. While absolute BMD would provide a more direct and linear assessment, *T*-scores were used to ensure consistency across measurements obtained in different clinical settings. This approach may have introduced some imprecision, particularly in the context of heterogeneous DXA devices, and should be considered when interpreting the magnitude of BMD changes.

Despite these limitations, the main strength is the consistency of the sCTX threshold associated with BMD maintenance at discontinuation between our study and Grassi et al. [24], even though the important methodologic differences between both.

### Clinical implications and conclusion

Apart from preventing rebound vertebral fractures, the BMD achieved with denosumab should ideally be maintained. Limiting denosumab duration may help reduce the rebound effect. In patients treated for longer, strategies should aim to minimize bone loss. Our findings support evidence that maintaining lower sCTX levels during the first-year post-discontinuation increases the likelihood of preserving BMD over 2 years. Our study suggests different sCTX thresholds for this objective. Targeting sCTX value with 100% sensitivity (129 ng/L in the first year) seems impossible in many patients. In the absence of evidence of an increased risk of vertebral fracture with a higher threshold, the target of 49% of ULN (281 ng/L) also proposed by ECTS, seems achievable. In our clinical practice, we initiate alendronate from the fourth month after the last denosumab administration to attenuate the rebound, followed by sCTX monitoring from the seventh month and then every two months. Switching to zoledronate is necessary depending on the sCTX threshold set and must be repeated at each follow-up during the first year if the target is exceeded [34]. Whether iterative versus single perfusions better limit BMD loss and fracture risk remains unstudied. In the absence of sufficient data, we think that iterative zoledronate infusions guided by careful monitoring of sCTX still appear necessary to prevent the worst outcomes in terms of BMD loss and fracture development.

Further research is warranted to determine whether increasing the dose of zoledronate or reducing the interval between infusions could more effectively control sCTX levels and mitigate BMD loss.

## Supplementary Information

Below is the link to the electronic supplementary material.

ESM 1 (PDF 237 KB)

## Data Availability

Data are available upon request.

## References

[1] Baron R, Ferrari S, Russell RGG (2011) Denosumab and bisphosphonates: different mechanisms of action and effects. Bone 48:677–692. 10.1016/j.bone.2010.11.020 21145999 10.1016/j.bone.2010.11.020

[2] McClung MR, Lewiecki EM, Cohen SB et al (2006) Denosumab in postmenopausal women with low bone mineral density. N Engl J Med 354:821–831. 10.1056/NEJMoa044459 16495394 10.1056/NEJMoa044459

[3] Bone HG, Wagman RB, Brandi ML et al (2017) 10 years of denosumab treatment in postmenopausal women with osteoporosis: results from the phase 3 randomised FREEDOM trial and open-label extension. Lancet Diabetes Endocrinol 5:513–523. 10.1016/S2213-8587(17)30138-9 28546097 10.1016/S2213-8587(17)30138-9

[4] Cummings SR, Martin JS, McClung MR et al (2009) Denosumab for prevention of fractures in postmenopausal women with osteoporosis. N Engl J Med 361:756–765. 10.1056/NEJMoa0809493 19671655 10.1056/NEJMoa0809493

[5] Anastasilakis AD, Yavropoulou MP, Makras P et al (2017) Increased osteoclastogenesis in patients with vertebral fractures following discontinuation of denosumab treatment. Eur J Endocrinol 176:677–683. 10.1530/EJE-16-1027 28283537 10.1530/EJE-16-1027

[6] McDonald MM, Khoo WH, Ng PY et al (2021) Osteoclasts recycle via osteomorphs during RANKL-stimulated bone resorption. Cell 184:1330-1347.e13. 10.1016/j.cell.2021.02.002 33636130 10.1016/j.cell.2021.02.002 PMC7938889

[7] Bone HG, Bolognese MA, Yuen CK et al (2011) Effects of denosumab treatment and discontinuation on bone mineral density and bone turnover markers in postmenopausal women with low bone mass. J Clin Endocrinol Metab 96:972–980. 10.1210/jc.2010-1502 21289258 10.1210/jc.2010-1502

[8] Aubry-Rozier B, Gonzalez-Rodriguez E, Stoll D, Lamy O (2016) Severe spontaneous vertebral fractures after denosumab discontinuation: three case reports. Osteoporos Int 27:1923–1925. 10.1007/s00198-015-3380-y 26510845 10.1007/s00198-015-3380-y

[9] Anastasilakis AD, Polyzos SA, Makras P et al (2017) Clinical features of 24 patients with rebound-associated vertebral fractures after denosumab discontinuation: systematic review and additional cases: DENOSUMAB DISCONTINUATION AND VERTEBRAL FRACTURES. J Bone Miner Res 32:1291–1296. 10.1002/jbmr.3110 28240371 10.1002/jbmr.3110

[10] McClung MR, Wagman RB, Miller PD et al (2017) Observations following discontinuation of long-term denosumab therapy. Osteoporos Int 28:1723–1732. 10.1007/s00198-017-3919-1 28144701 10.1007/s00198-017-3919-1 PMC5391373

[11] Zanchetta MB, Boailchuk J, Massari F et al (2018) Significant bone loss after stopping long-term denosumab treatment: a post FREEDOM study. Osteoporos Int 29:41–47. 10.1007/s00198-017-4242-6 28975362 10.1007/s00198-017-4242-6

[12] Burckhardt P, Faouzi M, Buclin T et al (2020) Fractures after denosumab discontinuation: a retrospective study of 797 cases. J Bone Miner Res 36:1717–1728. 10.1002/jbmr.4335 10.1002/jbmr.4335 PMC8518625 34009703

[13] Cosman F, Huang S, McDermott M, Cummings SR (2020) Multiple vertebral fractures after denosumab discontinuation: FREEDOM and FREEDOM extension trials additional post hoc analyses. J Bone Miner Res 37:2112–2120. 10.1002/jbmr.4705 10.1002/jbmr.4705 PMC10092421 36088628

[14] Lamy O, Gonzalez-Rodriguez E, Stoll D et al (2017) Severe rebound-associated vertebral fractures after denosumab discontinuation: 9 clinical cases report. J Clin Endocrinol Metab 102:354–358. 10.1210/jc.2016-3170 27732330 10.1210/jc.2016-3170

[15] Gonzalez-Rodriguez E, Aubry-Rozier B, Stoll D et al (2020) Sixty spontaneous vertebral fractures after denosumab discontinuation in 15 women with early-stage breast cancer under aromatase inhibitors. Breast Cancer Res Treat 179:153–159. 10.1007/s10549-019-05458-8 31598815 10.1007/s10549-019-05458-8

[16] Popp AW, Zysset PK, Lippuner K (2016) Rebound-associated vertebral fractures after discontinuation of denosumab—from clinic and biomechanics. Osteoporos Int 27:1917–1921. 10.1007/s00198-015-3458-6 26694598 10.1007/s00198-015-3458-6

[17] Everts-Graber J, Reichenbach S, Gahl B et al (2022) Effects of zoledronate on bone mineral density and bone turnover after long-term denosumab therapy: observations in a real-world setting. Bone 163:116498. 10.1016/j.bone.2022.116498 35882310 10.1016/j.bone.2022.116498

[18] Tsourdi E, Zillikens MC, Meier C et al (2020) Fracture risk and management of discontinuation of denosumab therapy: a systematic review and position statement by ECTS. J Clin Endocrinol Metab. 10.1210/clinem/dgaa756 33103722 10.1210/clinem/dgaa756

[19] Tsourdi E, Langdahl B, Cohen-Solal M et al (2017) Discontinuation of denosumab therapy for osteoporosis: a systematic review and position statement by ECTS. Bone 105:11–17. 10.1016/j.bone.2017.08.003 28789921 10.1016/j.bone.2017.08.003

[20] Sølling AS, Harsløf T, Langdahl B (2020) Treatment with zoledronate subsequent to denosumab in osteoporosis: a randomized trial. J Bone Miner Res 35:1858–1870. 10.1002/jbmr.4098 32459005 10.1002/jbmr.4098

[21] Sølling AS, Tsourdi E, Harsløf T, Langdahl BL (2023) Denosumab discontinuation. Curr Osteoporos Rep 21:95–103. 10.1007/s11914-022-00771-6 36564572 10.1007/s11914-022-00771-6

[22] Anastasilakis AD, Papapoulos SE, Polyzos SA et al (2019) Zoledronate for the prevention of bone loss in women discontinuing denosumab treatment. A prospective 2-year clinical trial. J Bone Miner Res 34:2220–2228. 10.1002/jbmr.3853 31433518 10.1002/jbmr.3853

[23] Liebich G, Lamy O, Aubry-Rozier B, Gonzalez-Rodriguez E (2023) Maintenance of bone resorption markers in the low premenopausal range during the year following denosumab discontinuation is associated to bone density preservation. The ReoLaus study. Bone 172:116764. 10.1016/j.bone.2023.116764 37062514 10.1016/j.bone.2023.116764

[24] Grassi G, Ghielmetti A, Zampogna M et al (2024) Zoledronate after denosumab discontinuation: is repeated administrations more effective than single infusion? J Clin Endocrinol Metab. 10.1210/clinem/dgae224 38609157 10.1210/clinem/dgae224 PMC11403318

[25] Everts-Graber J, Reichenbach S, Ziswiler HR et al (2020) A single infusion of zoledronate in postmenopausal women following denosumab discontinuation results in partial conservation of bone mass gains. J Bone Miner Res 35:1207–1215. 10.1002/jbmr.3962 31991007 10.1002/jbmr.3962

[26] Lehmann T, Aeberli D (2017) Possible protective effect of switching from denosumab to zoledronic acid on vertebral fractures. Osteoporos Int 28:3067–3068. 10.1007/s00198-017-4108-y 28589418 10.1007/s00198-017-4108-y

[27] Sølling AS, Harsløf T, Langdahl B (2020) Treatment with zoledronate subsequent to denosumab in osteoporosis: a 2-year randomized study. J Bone Miner Res 36:1245–1254. 10.1002/jbmr.4305 10.1002/jbmr.4305 33813753

[28] Makras P, Appelman-Dijkstra NM, Papapoulos SE et al (2021) The duration of denosumab treatment and the efficacy of zoledronate to preserve bone mineral density after its discontinuation. J Clin Endocrinol Metab 106:e4155–e4162. 10.1210/clinem/dgab321 33978745 10.1210/clinem/dgab321

[29] Everts-Graber J, Reichenbach S, Gahl B et al (2021) Risk factors for vertebral fractures and bone loss after denosumab discontinuation: a real-world observational study. Bone 144:115830. 10.1016/j.bone.2020.115830 33359006 10.1016/j.bone.2020.115830

[30] Farias V, Jerkovich F, Barragán AM et al (2024) Three-year effect of bisphosphonates on bone mineral density after denosumab withdrawal: observations from a real-world study. JBMR Plus 8:ziae044. 10.1093/jbmrpl/ziae044 38764789 10.1093/jbmrpl/ziae044 PMC11102571

[31] Eastell R, Christiansen C, Grauer A et al (2011) Effects of denosumab on bone turnover markers in postmenopausal osteoporosis. J Bone Miner Res 26:530–537. 10.1002/jbmr.251 20839290 10.1002/jbmr.251

[32] De Papp AE, Bone HG, Caulfield MP et al (2007) A cross-sectional study of bone turnover markers in healthy premenopausal women. Bone 40:1222–1230. 10.1016/j.bone.2007.01.008 17331821 10.1016/j.bone.2007.01.008

[33] Lamy O, Stoll D, Aubry-Rozier B, Rodriguez EG (2019) Stopping denosumab. Curr Osteoporos Rep 17:8–15. 10.1007/s11914-019-00502-4 30659428 10.1007/s11914-019-00502-4

[34] Lamy O, Everts-Graber J, Rodriguez EG (2025) Denosumab for osteoporosis treatment: when, how, for whom, and for how long. A pragmatical approach. Aging Clin Exp Res 37:70. 10.1007/s40520-025-02991-z 40055268 10.1007/s40520-025-02991-z PMC11889064

