# Supplementary material for: Keeping bone resorption markers very low for 1 year after denosumab prevents bone loss at 2 years: the ReoLaus study
Source: Arch Osteoporos. 2026 Aug 8;21(1):115. doi: 10.1007/s11657-026-01747-0 (PMC13452827; doi:10.1007/s11657-026-01747-0)
Supplement: Supplementary file 1 [file 11657_2026_1747_MOESM1_ESM.pdf]

Supplementary Information

Title : Keeping bone resorption markers very low for one year after denosumab prevents bone loss at two years. The ReoLaus study.

Journal : Osteoporosis International

Authors : Giovanni Liebich, Olivier Lamy, Jérôme Pasquier, Elena Gonzalez-Rodriguez.

Corresponding author : Elena Gonzalez Rodriguez, elena.gonzalez-rodriguez@chuv.ch

Supplementary table 1. BMD evolution

	**under denosumab**	**0-1, first year**	**1-2, second year**	**0-2, two years**
ΔTS LS, mean ± SD (n)	+0.9 ± 0.5 (107)	-0.3 ± 0.4 (116)	+0.0 ± 0.4 (103)	-0.2 ± 0.4 (114)
ΔTS FN, mean ± SD (n)	+0.3 ± 0.3 (101)	-0.1 ± 0.3 (110)	+0.0 ± 0.3 (102)	-0.1 ± 0.3 (111)
ΔTS TH, mean ± SD (n)	+0.4 ± 0.3 (106)	-0.1 ± 0.2 (118)	+0.0 ± 0.3 (103)	-0.1 ± 0.3 (114)

365-days standardized changes on T-score expressed in mean ± SD (n). during denosumab, on the first, on the second and on the two years after T0. T0 = 6 months after last denosumab

Supplementary table 2. Characteristics of losers and non-losers at femoral neck two years after the end of denosumab efficacity

**FN 02, n= 111**	**Losers, n= 23 (0.21)**	**Non-losers, n= 88 (0.79)**	**t-test**
	**Mean ± SD**	**Mean ± SD**	**p-value**
**Age**			
Dmab initiation, years	63.38 ± 10.05	65.49 ± 9.33	0.37
Dmab end, years	68.35 ± 9.79	69.52 ± 9.25	0.61
Time from menopause to Dmab, years	12.14 ± 8.68	14.74 ± 8.27	0.24
**BMI,** kg/m^2	23.05 ± 3.30	23.64 ± 3.61	0.45
**Dmab**			
Injections, number	9.04 ± 2.38	7.85 ± 2.99	0.05
duration, months	60.55 ± 15.65	49.05 ± 19.15	0.005
**DXA0**			
TS LS	-1.7 ± 1.4	-1.8 ± 1.1	0.94
TS FN	-1.4 ± 0.7	-1.7 ± 0.7	0.08
TS TH	-1.3 ± 0.7	-1.4 ± 0.8	0.53
TBS	1.300 ± 0.087	1.290 ± 0.098	0.66
**DXA1**			
TS LS	-2.3 ± 1.2	-2.1 ± 1.3	0.55
TS FN	-1.8 ± 0.7	-1.8 ± 0.7	0.99
TS TH	-1.5 ± 0.8	-1.6 ± 0.7	0.81
TBS	1.310 ± 0.068	1.308 ± 0.083	0.90
**DXA2**			
TS LS	-2.1 ± 1.5	-1.9 ± 1.2	0.55
TS FN	-1.9 ± 0.7	-1.7 ± 0.7	0.20
TS TH	-1.6 ± 0.8	-1.5 ± 0.8	0.34
TBS	1.290 ± 0.081	1.305 ± 0.084	0.43
**DXA changes 01 (first year)**			
ΔTS LS	-0.4 ± 0.3	-0.2 ± 0.4	0.02
ΔTS FN	-0.3 ± 0.2	-0.1 ± 0.2	<0.001
ΔTS TH	-0.2 ± 0.2	-0.1 ± 0.2	0.12
ΔTBS	0.009 ± 0.057	0.016 ± 0.048	0.68
**DXA changes 12 (second year)**			
ΔTS LS	-0.0 ± 0.5	0.1 ± 0.4	0.56

ΔTS FN	-0.1 ± 0.2	0.0 ± 0.2	0.004
ΔTS TH	-0.1 ± 0.3	0.1 ± 0.3	0.14
ΔTBS	-0.024 ± 0.048	-0.005 ± 0.049	0.15
**DXA changes 02 (two years)**			
ΔTS LS	-0.4 ± 0.5	-0.2 ± 0.4	0.09
ΔTS FN	-0.5 ± 0.2	0.0 ± 0.2	<0.001
ΔTS TH	-0.3 ± 0.3	-0.1 ± 0.2	<0.001
ΔTBS	-0.009 ± 0.049	0.012 ± 0.051	0.09
**sCTX 01 (first year)**			
AUC (% ULN)	92.7 ± 38.7	66.0 ± 37.9	0.007
**sCTX 12 (second year)**			
AUC (% ULN)	72.5 ± 25.9	59.0 ± 23.9	0.04
**sCTX 02 (two years)**			
AUC (% ULN)	83.7 ± 26.3	63.0 ± 28.4	0.004
	**Losers**	**Non losers**	**Fischer**
	**n (%)**	**n (%)**	**p-value**
**Risk Factors**			
Aromatase inhibitors, n	2 (9)	20 (23)	0.16
Other risk factors, n	3 (13)	15 (17)	0.76
**Fractured patients**			
**before dmab, n**	9 (39)	42 (48)	0.49
Vertebral Fractures, n	8 (35)	24 (27)	0.61
**during dmab, n**	2 (9)	6 (7)	0.67
Vertebral Fractures, n	2 (9)	3 (3)	0.28
**after dmab, n**	1 (4)	5 (6)	1.00
Vertebral Fractures, n	1 (4)	4 (5)	1.00
**OP treatment after dmab**			
BPs, n	22 (96)	85 (97)	1.00
Alendronate (ALN) only, n	0 (0)	8 (9)	0.20
Zoledronate (ZOL) only, n	14 (61)	53 (60)	1.00
ALN & ZOL mixed, n	8 (35)	22 (25)	0.43
1*ZOL, n	3 (13)	34 (39)	0.03
2*ZOL, n	14 (61)	29 (33)	0.02
≥3*ZOL, n	5 (22)	14 (16)	0.54
Time from last dmab to BP, months	2.4 ± 4.0	1.9 ± 2.9	0.58 (t-test)
**OP treatment before dmab**			
BPs, n	8 (35)	34 (39)	0.81
ALN, n	3 (13)	18 (20)	0.56
ZOL, n	1 (4)	6 (7)	1.00
Two or more different BPs, n	0 (0)	4 (5)	0.58
BPs duration, months	56.3 ± 62.9	62.0 ± 45.8	0.81 (t-test)
**BP before & after**	7 (30)	32 (36)	0.63
**Complementary treatment during dmab**	1 (4)	7 (8)	1.00

Dmab = denosumab; BMI = body mass index; BPs = bisphosphonates; ALN = alendronate; ZOL = zoledronate; TS = t-score; LS = lumbar spine; FN = femoral neck; TH = total hip; SD = standard deviation; m = month(s); T0 = time of rebound effect onset (6 months after last dmab injection); DXA = Dual X-ray Absorptiometry; DXA0 corresponds to the end of denosumab treatment (between 12 months before and 3.5 months after the end of denosumab efficacy); DXA1 was taken 12 months after T0 (8.5 to 15.5), and DXA2 24 months after T0 (20.5 to 36); 01 = first year; 12 = second year; 02 = two years; 365-days standardized DXA changes expressed in absolute t-score SD differences (ΔSD) for each site; TBS = Trabecular bone score; sCTX = serum crosslaps; AUC = area under the curve; %ULN = percentage of the upper limit of the norm for the pre-menopausal women; other risk factors include: long course systemic corticosteroid therapy (at least 3 months of prednisone equivalent doses of 5mg or more before denosumab), malabsorption syndrome (celiac disease, gastric bypass, bowel inflammatory diseases), primary hyperparathyroidism, rheumatoid arthritis, multiple sclerosis, monoclonal gammopathy and systemic mastocytosis. Values are expressed in number (%) or mean ± standard deviation (minimum, maximum).

Supplementary table 3. Characteristics of losers and non-losers in TBS two years after the end of denosumab efficacity

**TBS 02, n= 102**	**Losers, n= 21 (0.21)**	**Non-losers, n= 81 (0.79)**	**t-test**
	**Mean ± SD**	**Mean ± SD**	**p-value**
**Age**			
Dmab initiation, years	64.98 ± 11.84	64.98 ± 8.84	1.00
Dmab end, years	68.97 ± 11.41	69.18 ± 8.74	0.94
Time from menopause to dmab, years	14.62 ± 9.79	13.67 ± 8.14	0.71
**BMI,** kg/m^2	23.35 ± 3.73	23.42 ± 3.35	0.93
**Dmab**			
Injections, number	7.29 ± 2.87	8.15 ± 2.86	0.23
duration, months	48.53 ± 20.33	51.15 ± 18.38	0.60
**DXA0**			
TS LS	-1.8 ± 1.1	-1.7 ± 1.2	0.83
TS FN	-1.4 ± 0.7	-1.7 ± 0.7	0.13
TS TH	-1.1 ± 0.7	-1.4 ± 0.8	0.07
TBS	1.349 ± 0.078	1.281 ± 0.095	0.002
**DXA1**			
TS LS	-2.2 ± 1.1	-2.0 ± 1.3	0.63
TS FN	-1.7 ± 0.6	-1.8 ± 0.7	0.29
TS TH	-1.3 ± 0.7	-1.6 ± 0.7	0.07
TBS	1.307 ± 0.083	1.313 ± 0.083	0.78
**DXA2**			
TS LS	-2.0 ± 1.1	-1.9 ± 1.3	0.85
TS FN	-1.6 ± 0.8	-1.8 ± 0.8	0.40
TS TH	-1.1 ± 0.8	-1.5 ± 0.8	0.04
TBS	1.286 ± 0.079	1.310 ± 0.085	0.24
**DXA changes 01 (first year)**			
ΔTS LS	-0.2 ± 0.4	-0.2 ± 0.4	0.81
ΔTS FN	-0.1 ± 0.3	-0.1 ± 0.2	0.98
ΔTS TH	-0.0 ± 0.2	-0.2 ± 0.2	0.03
ΔTBS	-0.021 ± 0.053	0.025 ± 0.045	0.004
**DXA changes 12 (second year)**			
ΔTS LS	0.0 ± 0.3	0.0 ± 0.4	0.97
ΔTS FN	-0.1 ± 0.2	0.0 ± 0.2	0.005
ΔTS TH	0.0 ± 0.2	0.1 ± 0.3	0.52
ΔTBS	-0.036 ± 0.056	-0.001 ± 0.044	0.03
**DXA changes 02 (two years)**			
ΔTS LS	-0.2 ± 0.4	-0.2 ± 0.4	0.99
ΔTS FN	-0.2 ± 0.3	-0.1 ± 0.3	0.13
ΔTS TH	-0.0 ± 0.3	-0.1 ± 0.3	0.15
ΔTBS	-0.056 ± 0.016	0.024 ± 0.043	<0.001
**sCTX 01 (first year)**			
AUC (% ULN)	60.1 ± 24.2	75.0 ± 42.8	0.04
**sCTX 12 (second year)**			
AUC (% ULN)	61.7 ± 19.8	61.1 ± 27.0	0.91
**sCTX 02 (two years)**			
AUC (% ULN)	62.9 ± 18.5	68.2 ± 31.7	0.36
	**Losers**	**Non losers**	**Fischer**
	**n (%)**	**n (%)**	**p-value**
**Risk Factors**			
Aromatase inhibitors, n	3 (14)	15 (19)	0.76
Other risk factors, n	4 (19)	14 (17)	1.00
**Fractured patients**			
**before dmab, n**	8 (38)	44 (54)	0.23
Vertebral Fractures, n	6 (29)	26 (32)	1.00
**during dmab, n**	2 (10)	4 (5)	0.60
Vertebral Fractures, n	2 (10)	1 (1)	0.11

**after dmab, n**	1 (5)	4 (5)	1.00
Vertebral Fractures, n	1 (5)	3 (4)	1.00
**Osteoporotic treatment after dmab**			
BPs, n	20 (95)	79 (98)	0.50
Alendronate (ALN) only, n	1 (5)	8 (10)	0.68
Zoledronate (ZOL) only, n	12 (57)	51 (63)	0.62
ALN & ZOL mixed, n	7 (33)	19 (23)	0.40
1*ZOL, n	7 (33)	28 (35)	1.00
2*ZOL, n	9 (43)	27 (33)	0.45
≥3*ZOL, n	3 (14)	16 (20)	0.76
Time from last Dmab to BP, months	1.5 ± 1.7	2.3 ± 3.5	0.13 (t-test)
**Osteoporotic treatment before dmab**			
BPs, n	8 (38)	29 (36)	1.00
ALN, n	3 (14)	16 (20)	0.76
ZOL, n	1 (5)	5 (6)	1.00
Two or more different BPs, n	1 (5)	4 (5)	1.00
BPs duration, months	92.1 ± 81.6	50.6 ± 29.8	0.20 (t-test)
**BP before & after**	7 (33)	28 (35)	1.00
**Complementary treatment during dmab**	1 (5)	10 (12)	0.45

Dmab = denosumab; BMI = body mass index; BPs = bisphosphonates; ALN = alendronate; ZOL = zoledronate; TS = t-score; LS = lumbar spine; FN = femoral neck; TH = total hip; SD = standard deviation; m = month(s); T0 = time of rebound effect onset (6 months after last dmab injection); DXA = Dual X-ray Absorptiometry; DXA0 corresponds to the end of denosumab treatment (between 12 months before and 3.5 months after the end of denosumab efficacy); DXA1 was taken 12 months after T0 (8.5 to 15.5), and DXA2 24 months after T0 (20.5 to 36); 01 = first year; 12 = second year; 02 = two years; 365-days standardized DXA changes expressed in absolute t-score SD differences (ΔSD) for each site; TBS = Trabecular bone score; sCTX = serum crosslaps; AUC = area under the curve; %ULN = percentage of the upper limit of the norm for the pre-menopausal women; other risk factors include: long course systemic corticosteroid therapy (at least 3 months of prednisone equivalent doses of 5mg or more before denosumab), malabsorption syndrome (celiac disease, gastric bypass, bowel inflammatory diseases), primary hyperparathyroidism, rheumatoid arthritis, multiple sclerosis, monoclonal gammopathy and systemic mastocytosis. Values are expressed in number (%) or mean ± standard deviation (minimum, maximum).
